# Mitochondrial transfer in tunneling nanotubes—a new target for cancer therapy

**DOI:** 10.1186/s13046-024-03069-w

**Published:** 2024-05-21

**Authors:** Fan Guan, Xiaomin Wu, Jiatong Zhou, Yuzhe Lin, Yuqing He, Chunmei Fan, Zhaoyang Zeng, Wei Xiong

**Affiliations:** 1 NHC Key Laboratory of Carcinogenesis and Hunan Key Laboratory of Cancer Metabolism, Hunan Cancer Hospital and the Affiliated Cancer Hospital of Xiangya School of Medicine, Central South University, Changsha, China; 2 Key Laboratory of Carcinogenesis and Cancer Invasion of the Chinese Ministry of Education, Cancer Research Institute, Central South University, Changsha, China; 3 Department of Histology and Embryology, School of Basic Medicine Sciences, Central South University, Changsha, Hunan Province 410013 China

**Keywords:** Mitochondrial transfer, Oxidative phosphorylation, Metabolic symbiosis, Tunneling nanotubes, Immune evasion, Chemotherapy resistance, CAR-T

## Abstract

A century ago, the Warburg effect was first proposed, revealing that cancer cells predominantly rely on glycolysis during the process of tumorigenesis, even in the presence of abundant oxygen, shifting the main pathway of energy metabolism from the tricarboxylic acid cycle to aerobic glycolysis. Recent studies have unveiled the dynamic transfer of mitochondria within the tumor microenvironment, not only between tumor cells but also between tumor cells and stromal cells, immune cells, and others. In this review, we explore the pathways and mechanisms of mitochondrial transfer within the tumor microenvironment, as well as how these transfer activities promote tumor aggressiveness, chemotherapy resistance, and immune evasion. Further, we discuss the research progress and potential clinical significance targeting these phenomena. We also highlight the therapeutic potential of targeting intercellular mitochondrial transfer as a future anti-cancer strategy and enhancing cell-mediated immunotherapy.

**Graphical Abstract:**

**Figure:**
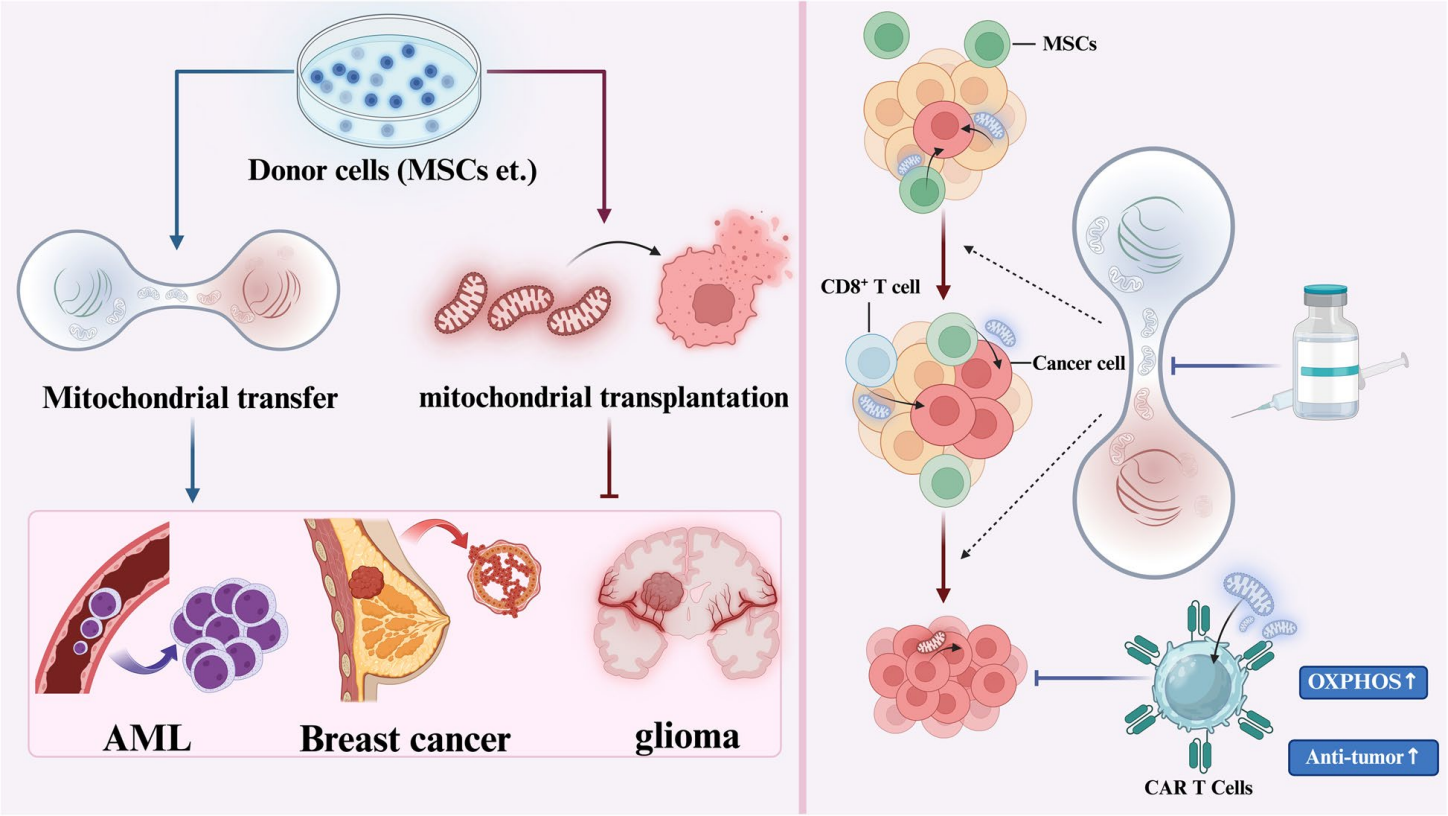
Mitochondrial Transfer in the Tumor Microenvironment. This review elaborates in detail on the molecular mechanisms and pathophysiological significance behind the mitochondrial transfer occurring between tumor cells and their microenvironment. This biological phenomenon of mitochondrial transfer is prevalent in a variety of cancers, including both solid tumors and hematologic malignancies, with a high incidence in acute myeloid leukemia (AML), breast cancer, and gliomas. The review also discusses therapeutic approaches targeting mitochondrial transfer, with a special focus on its application in immunotherapy, particularly in CAR-T cell therapy, where it has begun to show unique advantages

## Background

Unrestricted rapid proliferation is one of the most important biological characteristics of tumor cells [1]. To meet the substantial energy and nutritional needs for their rapid division, proliferation, and growth, tumor cells often exhibit a unique mode of energy metabolism. Even in the presence of sufficient oxygen, tumor cells prefer to metabolize glucose via glycolysis rather than through mitochondrial oxidative phosphorylation (OXPHOS); the latter produces more ATP. This phenomenon is known as aerobic glycolysis, also referred to as the Warburg effect, and is a main feature distinguishing tumor cells from normal cells [2]. Mitochondria are the centers of energy metabolism in all eukaryotes [3]. Studies have shown that some tumor cells indeed exhibit varying degrees of irreversible mitochondrial functional damage. For example, the activity of respiratory chain complex III in breast cancer cells is significantly lower compared to normal breast cells [4]. Other studies indicate that the main driving factor behind the high glycolysis of tumor cells is not mitochondrial dysfunction but rather an adaptive response to microenvironmental changes and genetic regulation. For instance, the activation of the c-Myc gene drives the increased expression of key glycolytic enzymes such as hexokinase (HK), pyruvate kinase M2 (PKM2), and glucose transporter 1 (GLUT-1). Meanwhile, the activation of protein kinase B (AKT) further triggers the activation of mTOR and the phosphorylation of HK and phosphofructokinase (PFK), while the inactivation of p53 upregulates the level of aerobic glycolysis [5–7]. Utilizing specific enzyme inhibitors, such as lactate dehydrogenase inhibitors, can slow down the conversion of pyruvate to lactate during aerobic glycolysis. When this conversion is reduced, the function of mitochondrial OXPHOS may be restored, suggesting that mitochondrial function has not been lost but has entered a specific "dormant" state.

However, recent studies have shown that mitochondria and the mitochondrial genome (mitochondrial DNA (mtDNA)) also play significant roles in tumor cells and their metabolic microenvironment. For instance, certain mitochondrial metabolites are sufficient to drive tumorigenesis [8]; some mitochondrial pathways adapt to the unique bioenergetic or biosynthetic metabolic functions of tumors, thereby endowing malignant cells with considerable metabolic plasticity [9, 10]. Interestingly, it has been further discovered that mitochondria can undergo transfer within the tumor microenvironment [11], and this transfer is not only limited to tumor cells themselves [12, 13], but also occurs between tumor cells and stromal cells [14, 15], tumor cells and immune cells [16, 17], tumor cells and endothelial cells [18], or between tumor cells and homologous normal cells [19]. Moreover, mitochondrial transfer occurs between tumor cells and platelets [20]. In most cases, tumor cells plunder mitochondria from other cells in the microenvironment to promote their own invasion and metastasis [20, 21], chemotherapy resistance [22], and immune evasion [17]. In a few cases, tumor cells transfer mitochondria to stromal cells to resist oxidative stress [23]. High-metastasis tumor cells can also transfer mtDNA with metastatic mutations to low-metastasis cancer cells and stromal cells through small extracellular vesicles (s-EVs) [24]. In this review, we thoroughly explore the impact of the tumor microenvironment on mitochondrial dynamics and their transfer pathways and mechanisms, as well as the pathophysiological importance of these processes in the metabolic adaptation of tumor cells. Additionally, this article discusses potential clinical intervention strategies targeting the biological process of mitochondrial transfer and highlights its potential in future cancer treatments and enhancing the efficacy of cell-mediated immunotherapy.

## Mechanisms of mitochondrial transfer

### Tunneling nanotubes (TNTs)

Tunneling nanotubes represent the most crucial pathway for mitochondrial transfer between tumor cells. TNTs are nano-scale membranous channels between cells, with diameters ranging from 50 to 1500 nm and lengths of 5–200 μm, with some TNTs reaching thicknesses up to 700 nm [25]. TNTs are not empty membranous tubes but are filled with cytoskeletal filaments. F-actin is present in most TNTs and is a critical structural component. On one hand, the cross-linking of F-actin ensures the rigidity of the TNT, ensuring its stability for outward growth [26, 27]; on the other hand, the cross-linking of F-actin mediates the biosynthesis of TNTs and allows mitochondria to be transported along the cytoskeletal structures within TNTs [28].

Rustom and colleagues observed the ultrastructure of rat pheochromocytoma (PC) 12 cells, where they first discovered TNTs and observed intercellular material exchange via TNTs using organelle-specific fluorescent dyes [29]. Subsequently, studies using electron microscopy revealed that TNTs could transfer mitochondria from mesenchymal stem cells (MSCs) to cardiomyocytes, marking one of the earliest pieces of research to reveal TNT-mediated mitochondrial transfer between cells [11, 30]. With the advancement of imaging technologies, scanning electron microscopy (SEM), atomic force microscopy (AFM), cryo-electron microscopy, and others have been widely applied to observe TNTs [31]. The challenges posed by the fragility and sensitivity of TNTs have been overcome, and TNTs are now extensively studied. Currently, TNT-mediated mitochondrial transfer is involved in various cellular microenvironments, including the cardiovascular system, immune system, respiratory system, corneal epithelium, tumors, and in neuronal injury and neurodegeneration within the central nervous system [11]. TNTs participate in a wide range of physiological and pathological events, such as immune responses, cell proliferation and apoptosis, substance transport, and angiogenesis. TNTs can transport a variety of materials over distances as long as 150 mm, including mitochondria and other organelles, lipid droplets, proteins, ions, RNA, and pathogens [29, 32].

Two mechanisms of TNT formation have been extensively discussed [33, 34]. In the actin-driven mechanism, a cell extends a pseudopod-like protrusion containing actin filaments that fuses with the edge of a neighboring cell, leading to TNT formation. This mechanism does not rely on cell motility or close contact. The other mechanism is the cell displacement mechanism, which occurs when two closely situated cells move in opposite directions. During this movement, the cells extend membrane protrusions that lead to TNT formation, thus it highly depends on cell movement. Since continuous cell contact occurs within minutes, this process can be temporarily regulated [30, 35]. However, it is still unclear whether these two mechanisms lead to the same tubular structures and whether cells utilize both mechanisms simultaneously to form TNTs.

Specifically, the transport of mitochondria via TNTs involves mainly two mechanisms, one of which is primarily mediated by the mitochondrial Rho GTPase 1 (Miro1). Research has shown that TNTs mediate the transfer of mitochondria from MSCs to epithelial cells. If the Miro1 in MSCs is knocked out, mitochondria are unable to transfer to epithelial cells. This study confirmed that Miro1, acting as a motor protein-associated, calcium-sensitive linker protein, can mediate the transfer of mitochondria through TNTs from MSCs to epithelial cells, with Miro1 playing a role in regulating mitochondrial homeostasis and transport [36]. The specific mechanism involves Miro1, along with auxiliary proteins Miro2, transport associated protein 1 (TRAK1), TRAK2, and myosin XIX (Myo19), driving the mitochondria to bind with the Kruppel-like factor 5 (KLF5) driven proteins. When bound, they form a motor-adaptor complex, thereby facilitating the transport of mitochondria within TNTs and regulating their movement. At the same time, these structures stabilize and protect mitochondria from degradation.

### Extracellular vesicles (EVs)

In addition to transport mediated by tunneling nanotubes, mitochondria can also be transferred between cells encapsulated within EVs. EVs are nano-sized bilayer vesicles secreted by cells, capable of carrying various lipids, proteins, RNA, microRNA (miRNA), and mitochondria [37]. EVs include exosomes with a diameter of 30–100 nm, microvesicles ranging from 100 nm to 1 μm, and apoptotic bodies sized from 1 μm to more than 2 μm [38]. Due to their smaller volume, exosomes can only carry mtDNA, miRNA, cytokines, chemokines, and other small molecular proteins, while microvesicles are capable of transporting entire mitochondria [39].

### Artificial mechanisms of mitochondrial transfer

Aside from the physiological release and uptake of mitochondria, artificial transfer of mitochondria has also been achieved. Researchers have developed a technique called MitoCeption, which relies on the ability of recipient cells to internalize isolated mitochondria, a process associated with macropinocytosis [40]. Labeled mitochondria isolated from donor cells are centrifuged together with similarly labeled recipient cells, then co-cultured under normal conditions. During co-culture, the isolated mitochondria are internalized by the recipient cells, thereby allowing entry into the recipient cells [41]. The process of mitochondria entering recipient cells through internalization induced by centrifugation and heat shock during co-culture is referred to as MitoCeption. The researchers developed MitoCeption to achieve mitochondrial transfer from MSCs to cancer cells. They further validated the mitochondrial transfer process using microscopy imaging, fluorescence-activated cell sorting (FACS), real-time quantitative PCR (qPCR) and other techniques [42]. Techniques like MitoCeption, which represent artificial mitochondrial transfer, not only provide effective tools for studying intercellular mitochondrial transfer but also have significant implications for understanding cell interactions within the tumor microenvironment, developing new therapeutic strategies, and studying the role of mitochondria in diseases.

### Cell fusion

Cell fusion can also mediate the transfer of mitochondria between cells. This process occurs both through partial cell fusion facilitated by TNTs and through complete cell fusion. Researchers co-cultured free mitochondria extracted from cervical cancer cells with cardiomyocytes and observed the mitochondria being endocytosed by the cardiomyocytes [43]. However, when the actin polymerization of cardiomyocytes was inhibited using cytochalasin D (CytoD), the transfer of mitochondria to cardiomyocytes was significantly reduced [44]. This study represents a process of partial cell fusion achieved through TNTs. In the case of complete cell fusion, mitochondrial transfer can be identified when MSCs are co-cultured with transgenic mouse cardiomyocytes and fully fuse with mitochondria-damaged cardiomyocytes. However, since cell fusion is rarely found in higher eukaryotes under normal physiological conditions, it is not the main mechanism for mitochondrial transfer.

**Fig. 1:**
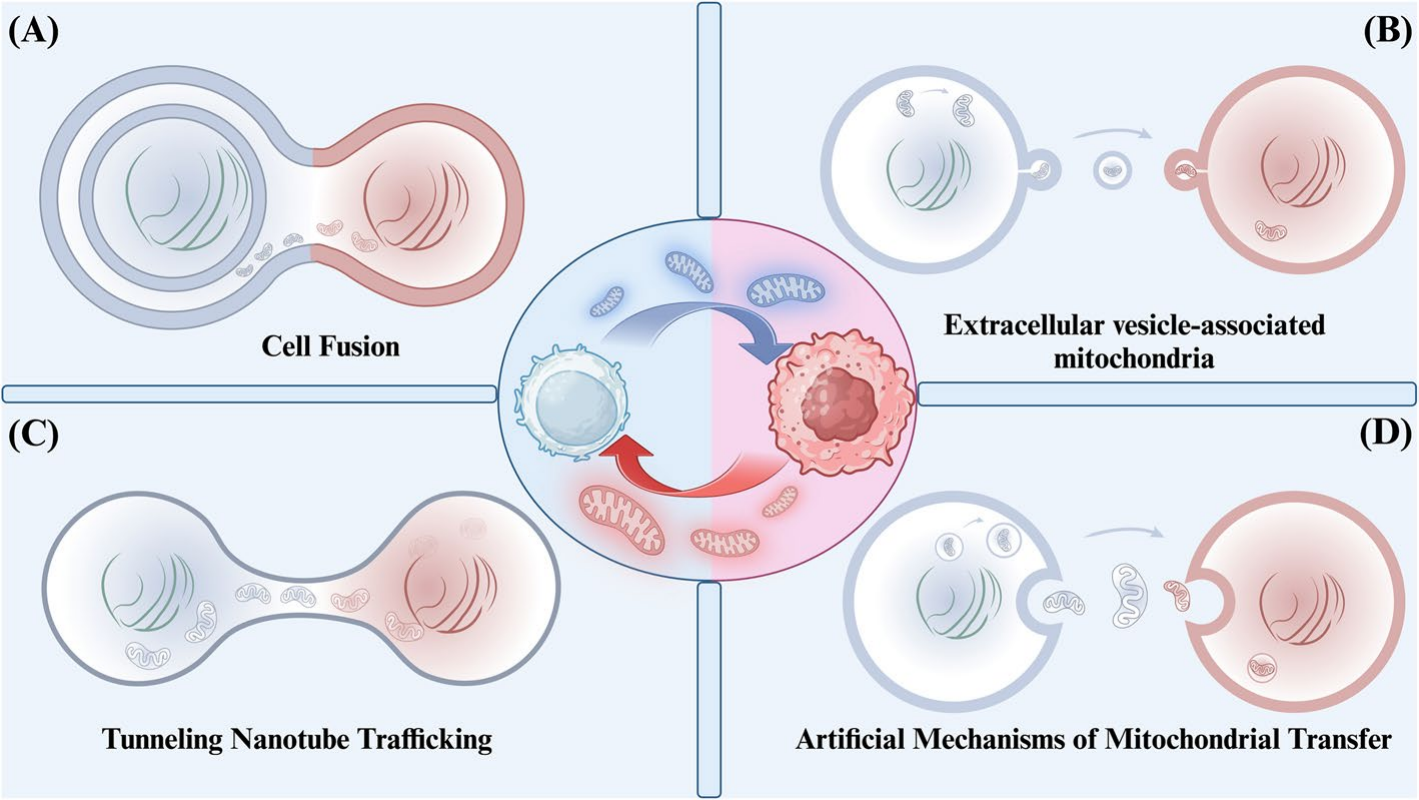
Modes of mitochondrial transfer in the tumor microenvironment

Current research has revealed that cells release free mitochondria in a manner dependent on mitochondrial fission proteins, such as dynamin-related protein 1 (DRP1) and mitochondrial fission 1 protein (FIS1). However, more studies are needed to fully understand the specific mechanisms through which mitochondria are packaged and transported (Fig. 1).

## Mitochondrial transfer in tumor dynamics

### Mitochondrial transfer promotes tumor proliferation

**Fig. 2:**
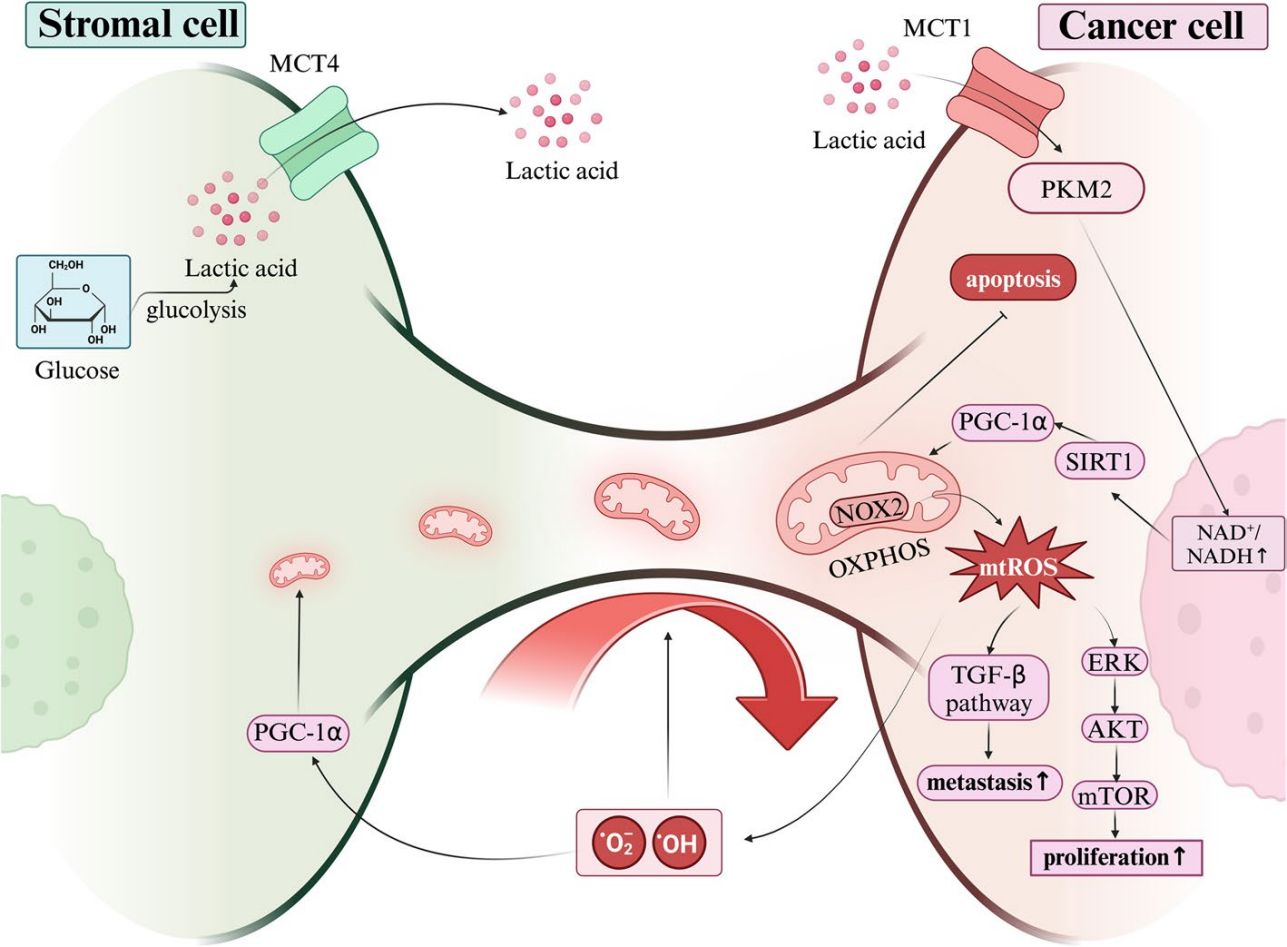
Mitochondrial transfer from stromal cells to tumor cells. In AML cells, NOX2 in mitochondria can produce ROS to further promote tumor cell proliferation. The possible mechanism is that mitochondrial Reactive Oxygen Species (mtROS) stimulates ERK to further activate the AKT-mTOR signaling pathway. The mtROS generated by mitochondria can also activate the transforming growth factor- β (TGF-β) signaling pathway, thereby promoting tumor metastasis. In prostate cancer, tumor cells change the metabolic pattern of cancer-associated fibroblasts (CAFs), causing them to produce lactate. Then, through their own lactate receptor monocarboxylate transporter 1(MCT1), the lactate is received, further activating PKM2 into the nucleus, and enhancing the demand for CAF mitochondria through the downstream SIRT1-PGC-1α signaling pathway

The earliest studies showed that ρ0 tumor cells, which lack mtDNA, divided more slowly [11], but could restore respiratory function by seizing whole mitochondria and associated mtDNA from neighboring cells [45–47], thereby achieving faster cell division and restoring their tumorigenic capacity [46, 48]. Research indicates that unlike solid tumors, AML cells rely more on OXPHOS than on glycolysis [49]. In AML cells, NADPH oxidase 2 (NOX2) generates superoxides, stimulating bone marrow stromal cells (BMSCs) to transfer mitochondria to AML blast cells via TNTs derived from AML, thereby promoting AML cell proliferation. However, NOX2 has no detectable effect on the survival of non-malignant CD34^+^ cells, suggesting AML cells may specifically plunder mitochondria from stromal cells via NOX2 to meet their proliferation needs [50]. Interestingly, another study showed that transferred mitochondria are not in optimal condition but rather are dysfunctional with high Reactive Oxygen Species (ROS) content, and recipient tumor cells do not require these dysfunctional mitochondria to restore their respiratory function but rather stimulate other signaling pathways through ROS. For example, malignant breast cancer cells plunder dysfunctional mitochondria from macrophages in the microenvironment and accumulate ROS, which activates the ERK pathway thus promoting tumor proliferation [51] (Fig. 2). In gliomas, the tumor-associated stromal cells (TASCs) transfer mitochondria to glioblastoma (GBM) cells, thereby promoting tumor proliferation. This transfer occurs through structures such as TNTs, EVs, etc [52]. Studies have shown that lung cancer cells form cancer-initiating cell (CIC) structures to plunder lymphocyte mitochondria, thereby activating the mitogen-activated protein kinase (MAPK) and AKT signaling pathways, promoting their own proliferation [17]. The transfer of mitochondria from stem cells to immortalized cells rapidly induces tumorigenesis. For instance, the transfer of mitochondria from adipose stem cells to HEK293 cells not only makes them more tumorigenic but also enhances their aggressiveness [53]. In gliomas, the uptake of astrocyte mitochondria promotes cell cycle progression to the proliferative G2/M phase, enhancing self-renewal and tumorigenicity of GBM cells [54, 55]. Some research teams have developed a tool, MitoCeption (as mentioned above), that enables the transfer of mitochondria in stromal cell to tumor cells, thereby restoring respiratory function and increasing proliferation rates [42]. In melanoma, tumor cells attract bone marrow-derived stromal cells (MSCs) to the primary tumor site, stimulate PGC-1α to promote the biogenesis of MSC mitochondria, and transfer these mitochondria to tumor cells to promote their proliferatio [56]. The mechanism by which exogenous mitochondria drive cancer cell proliferation is not clear but may be related to the production of ROS [51], and ROS can stimulate the transport of mitochondria to cancer cells [50].

### Mitochondrial transfer promotes tumor invasion and metastasis

**Fig. 3:**
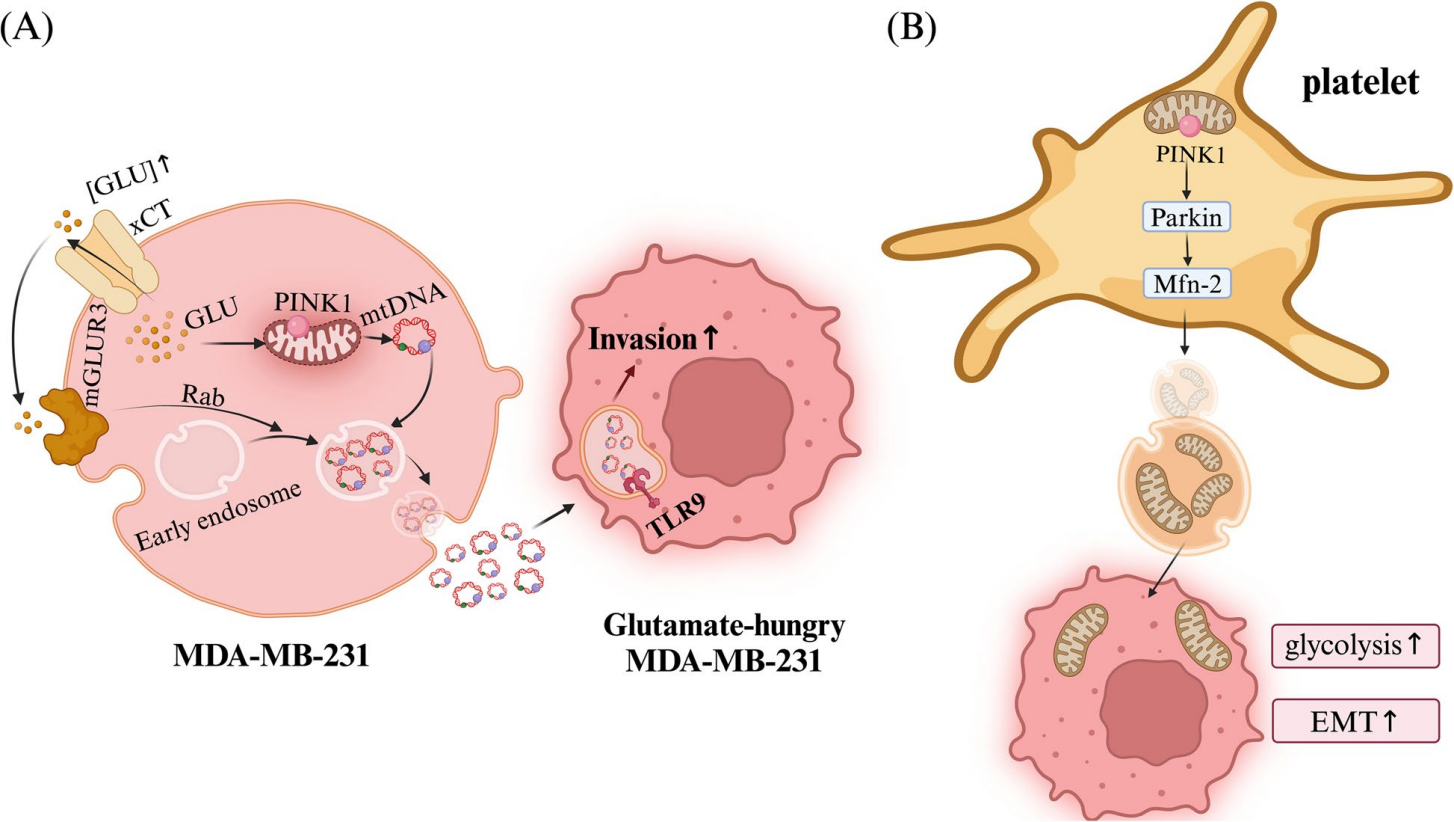
Mitochondria and their mtDNA are transferred via EVs. **A** In the glutamate in MDA-MB-231 cells is encapsulated into EVs through the PINK1 pathway, leading to an increase in extracellular glutamate concentration which activates mGLUR3 on the cell membrane. This activation stimulates the downstream Rab, promoting the packaging and transport of mtDNA. The mtDNA released from cells is reuptaken by glutamate-hungry MDA-MB-231, which, by stimulating TLR9, enhances the invasive capability of glutamate-hungry MDA-MB-231. **B** In platelets transfer mitochondria to tumor cells via the PINK1/Parkin-Mfn2 pathway via EVs. The mitochondria from platelets promote an increase in glycolytic metabolism and a decrease in ROS levels in tumor cells, although their proliferation rate decreases. As a result, they are more susceptible to experiencing EMT, which can consequently lead to metastasis

**Fig. 4:**
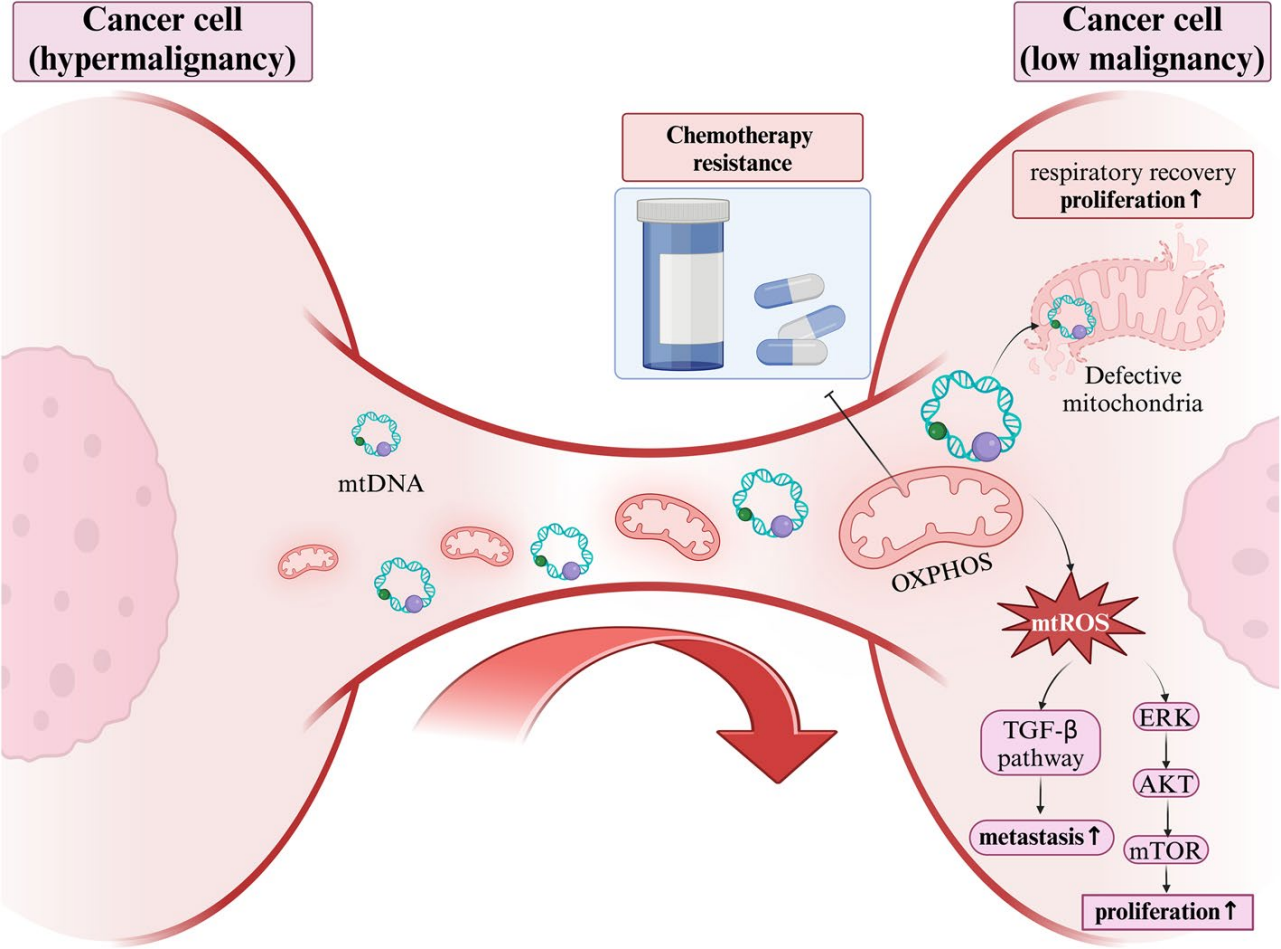
Highly malignant tumor cells can transfer mitochondria to less malignant tumor cells. The transferred mitochondria increases the proliferation and metastatic capabilities of the less malignant tumor cells. Moreover, mtDNA can also be transferred, which restores the dysfunctional mitochondria in less malignant tumor cells, enabling them to regain respiratory function. In addition, tumor cells resistant to chemotherapy transfer mitochondria to chemotherapy-sensitive tumor cells, making them resistant to chemotherapy

Increasing evidence indicates that mitochondrial transfer promotes the invasion and metastasis of tumor cells, which is one of the main hallmarks of tumors. In prostate cancer (PCa) research, it has been discovered that tumor cells and CAFs form a dual-compartment metabolic system that promotes the malignant progression of cancer [57, 58]. In this interaction, CAFs change their metabolism to glycolysis after contact with PCa, producing lactate and releasing it into the surrounding microenvironment. PCa cells absorb these lactates through the MCT1 on their surface, further triggering the nuclear translocation of PKM2, which acts as a transcription factor participating in and activating OXPHOS [59], changing the intracellular NAD^+^/NADH ratio, and then enhancing mitochondrial activity in PCa cells through the SIRT1—PGC-1α pathway. To meet this increased energy demand, CAFs transfer mitochondria to PCa cells through TNTs. This metabolic mode is also known as the "Reverse Warburg Effect" [60]. In another study, overexpression of mitochondrial fission factor (MFF) in an hTERT-immortalized human fibroblast line led to oxidative stress, increased ROS production, NF-kB activation, driving autophagy, mitophagy, and ultimately glycolytic metabolism, resulting in mitochondrial dysfunction. MFF-overexpressing fibroblasts similarly exhibited increased ATP consumption and L-lactate production, thus promoting tumor development [61]. This metabolic symbiosis not only enhances the energy metabolism of PCa cells but also promotes tumor invasiveness and epithelial-mesenchymal transition (EMT) [14], which is an important step for cancer cells to increase mobility and invasiveness. Research shows that mitochondrial transfer occurs between tumor cells, and the core component of mitochondria, mtDNA, can also be transferred individually. The increase of extracellular glutamate through mGluR3 activation in MDA-MB-231 (a human breast cancer cell line) leads to the release of EVs dependent on Rab27. These EVs contain mtDNA packaged through a PTEN-induced putative kinase 1 (PINK1) -dependent manner, are transported to glutamate-starved MDA-MB-231 cells, and enhance their invasiveness and metastatic capacity through cell surface toll-like receptor 9 (TLR9) [13](Fig. 3). Direct transfer of mitochondria to MDA-MB-231 cells can enhance their proliferative and invasive capabilities [62], and also increase their sensitivity to cisplatin [63]. Studies have shown that melanoma cells plunder mtDNA from host cells to restore respiratory capacity, promoting their own invasion and metastasis. The inability of tumor cells to fully restore mitochondrial function during prolonged ex vivo maintenance highlights a crucial aspect. These cells require exposure to the microenvironment to regain their complete respiratory capacity. This underscores the significance of mitochondrial transfer and its components within the tumor microenvironment for tumor development and progression [48]. Subsequent studies by the same authors proved that tumor formation directly depends on the restoration of mitochondrial respiration [64]. Research found that high activity of the TCA cycle or electron transport chain (ETC) can activate the TGF-β pathway through mtROS, promoting cancer migration, invasion, and metastasis. This might be one of the important reasons why tumor cells plunder mitochondria to promote their invasion and metastasis [65]. In bladder cancer, mitochondrial transfer between heterogeneous tumor cells occurs (Fig. 4). Fluorescence imaging and flow cytometry detected the spontaneous unidirectional transfer of mitochondria from T24 to RT4 cells, and the AKT, mTOR, and downstream mediators were activated and increased in recipient cells. An increase in the invasiveness of bladder cancer cells was detected both in *vitro* and in *vivo* [12]. Other studies have shown that mitochondria from platelets transferred to tumor cells through the PINK1/Parkin-Mfn2 pathway, increasing their dependency on glycolytic metabolism to control intracellular ROS levels, and reducing their proliferation rate. However, this process promotes EMT in tumor cells, leading to metastasis [20].

### Mitochondrial transfer helps tumor cells cope with oxidative stress

Whether mitochondria are transferred into or out of tumor cells, there are instances showing that this transfer reduces the oxidative stress faced by tumor cells, thereby aiding tumor development and progression. PC12 cells avoid apoptosis induced by ultraviolet (UV) radiation by plundering mitochondria from healthy cells [66]. The transfer of mitochondria from MSC cells to ALL cells reduces the mortality of ALL cells after treatment with ROS and subsequent treatment with cytarabine (AraC) and daunorubicin (DNR) [15]. Studies have shown that the transfer of mitochondria from platelets to tumor cells shifts their metabolic mode towards glycolysis, and by controlling the ratio of oxidized glutathione to ROS within tumor cells, it effectively protects them. Destroying platelet mitochondria or inhibiting this antioxidant pathway increases the apoptosis rate of tumor cells [20]. In response to oxidative stress, tumor cells can also transfer mitochondria to stromal cells. Research indicates that T-ALL cells transfer mitochondria to mesenchymal stem cells via cell adhesion to reduce the oxidative stress produced by chemotherapy, with few mitochondria being accepted from mesenchymal stem cells [23]. This demonstrates that the transfer of mitochondria between tumor cells and stromal cells is often bidirectional.

### Mitochondrial transfer promotes tumor resistance to chemotherapy

Chemotherapy remains one of the most common means of controlling and treating cancer clinically, yet many cancers exhibit chemotherapy resistance during clinical treatment [67, 68]. Interestingly, one of the most common phenotypes gained by tumor cells through mitochondrial transfer is chemotherapy resistance. This phenotype was first observed in 2013 when MCF-7 cancer cells, having acquired mitochondria from epithelial cells, gained resistance to doxorubicin [18]. Studies suggest that mitochondrial transfer may occur in response to chemical drugs posing a threat to tumor survival. For example, multiple myeloma cells primarily metabolize through glycolysis [69] and have been shown to be sensitive to glycolysis inhibitors [70, 71]. However, it was found that multiple myeloma cells could perform functional OXPHOS after treatment with the glycolysis inhibitor ritonavir, and mitochondria were transferred from BMSCs to multiple myeloma cells when co-cultured together. Using a CD38-targeting agent (which inhibits the formation of TNTs) in combination with a glycolysis inhibitor led to extensive apoptosis of malignant plasma cells. This suggests that multiple myeloma cells resist chemical drugs by plundering mitochondria from stromal cells [72, 73]. It has also been shown that multiple myeloma cells can resist belamaf (a monoclonal antibody conjugated with microtubule-disrupting agent monomethyl auristatin-F (MMAF)) through this mechanism, thus avoiding apoptosis [74].

In ALL, mitochondrial transfer occurs more frequently, and traditional chemotherapy drugs like AraC and DNR always fail to completely eliminate tumor cells [75]. A more thorough clearance of ALL cells was achieved by using microtubule inhibitors (e.g., vincristine (VCR)) to disrupt the formation of TNTs, which could hinder the transfer of mitochondria from MSCs to ALL cells [15]. Under chemotherapy pressure, tumor cells can also transfer mitochondria to stromal cells to avoid being killed [23]. Further research indicates that treating AML cells with OXPHOS inhibitors rapidly induces both the transfer of exogenous mitochondria from bone marrow (BM) stromal cells to AML cells and the internal mitochondrial fission and mitophagy. This spontaneous enhancement of mitochondrial quality and quantity in tumor cells plays a crucial role in the compensatory adaptation of AML cells to energy stress in the BM environment [76–78]. Mitochondrial transfer from mesenchymal stem cells through metabolic rewiring also endows glioblastoma stem cells with chemotherapy resistance [52, 79]. Additionally, chemotherapy-resistant triple-negative breast cancer (TNBC) has been shown to transport mitochondria with mutant genes (mtND4) to ordinary TNBC cells via EVs, thereby conferring chemotherapy resistance [80]. The transfer of mtDNA levels from EVs also acts as a carcinogenic signal, promoting the exit of treatment-induced cancer stem-like cells from dormancy and leading to endocrine therapy resistance in OXPHOS-dependent breast cancer [81]. Mitochondrial transfer also occurs between tumor cells and endothelial cells, a pathway that is preferred over the formation between tumor cells and stromal cells, and also modulates chemotherapy resistance in tumor cells [18].

### Mitochondrial transfer promotes tumor immune escape

**Fig. 5:**
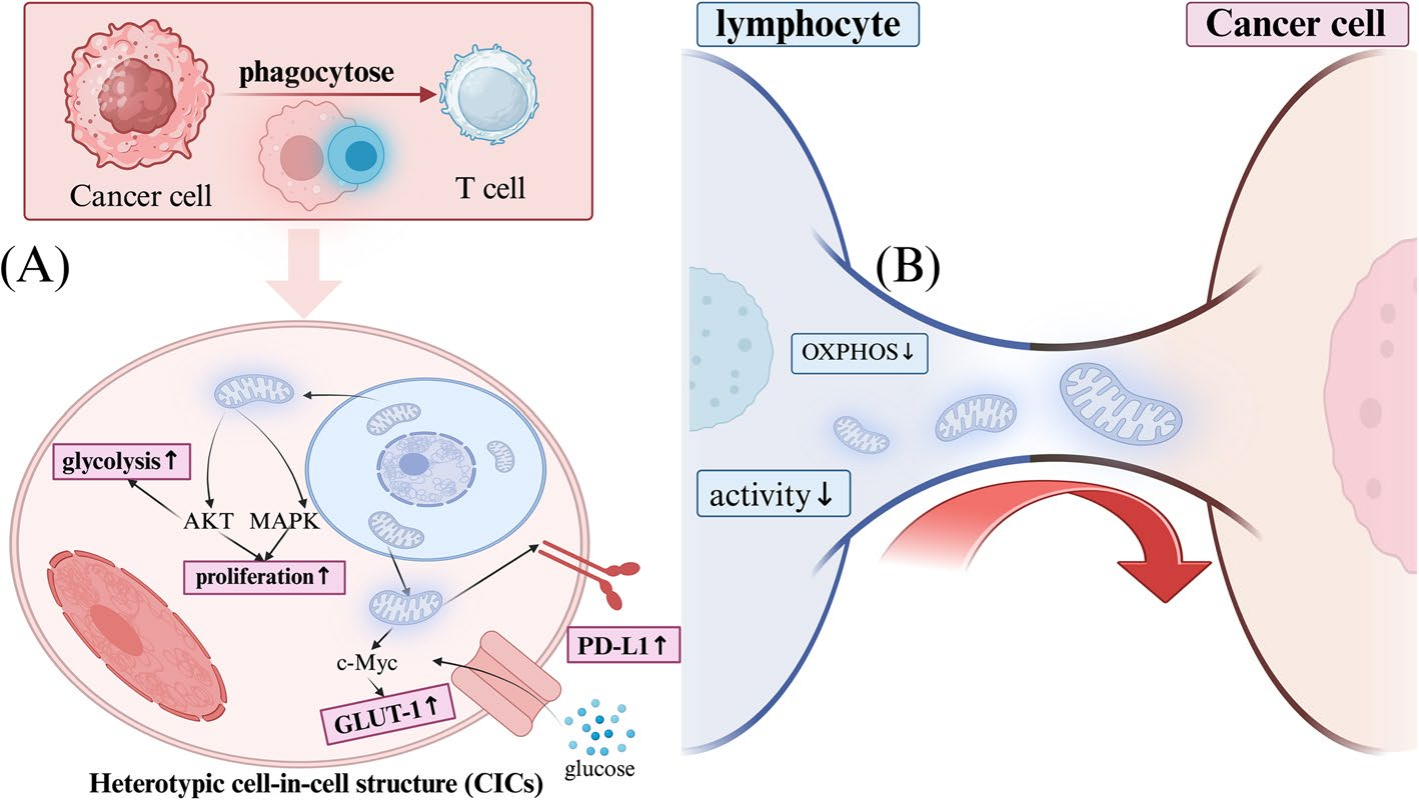
Mitochondrial transfer from CD8^+^T cells to tumor cells. **A** Tumor cells hijack mitochondria from immune cells to facilitate immune evasion. Tumor cells can engulf T cells into their cytoplasm to form heterotypic cell-in-cell structures (CICs). The mitochondria from T cells entering tumor cells can promote tumor cell proliferation through the AKT and MAPK signaling pathways and upregulate their glycolytic metabolism process. It can also activate c-Myc to increase the expression of GLUT-1, thereby enhancing glucose uptake. Most importantly, by hijacking mitochondria from T cells, tumor cells upregulate PD-L1 on the cell membrane. **B** T cells also transfer mitochondria to tumor cells through TNTs, leading to a reduction in their own OXPHOS levels, a decrease in anti-tumor immunity, and promoting tumor immune evasion

Mitochondrial transfer, as a significant biological phenomenon, has recently been revealed to play a key role in promoting tumor immune evasion, offering a new perspective on how tumors evade immune surveillance. Cell-in-cell structures (CICs) is defined as the entry of living cells of one type into another type of cell [82], and studies have found that CICs between immune cells and tumor cells are associated with the malignancy of many cancers. It has been shown that lung cancer cells form CICs with infiltrating lymphocytes, and through these CICs, mitochondria are transferred from lymphocytes to tumor cells, promoting immune evasion by upregulating PD-L1 expression (Fig. 5). CICs also induce reprogramming of glucose metabolism in lung cancer cells by upregulating glucose intake and glycolytic enzymes, affecting the normal energy metabolism of lymphocytes and weakening their immune killing ability [17]. Further studies using emission scanning electron microscopy, fluorescently labeled mitochondrial transfer tracking, and metabolic quantification have directly demonstrated that mitochondrial transfer from immune cells to cancer cells mediated by TNTs metabolically enhances cancer cells and depletes immune cells. Inhibiting the assembly mechanism of nanotubes can significantly reduce mitochondrial transfer and prevent immune cell exhaustion [16]. In the latest research, single-cell RNA sequencing technology combined with mitochondrial-enabled reconstruction of cellular interactions (MERCI) was used to track and quantify mitochondrial traffic between cancer cells and T cells. The application of MERCI to human cancer samples identified recurrent phenotypes associated with mitochondrial transfer, with characteristic genes involved in cytoskeletal remodeling, energy production, and the tumor necrosis factor-α (TNF-α) signaling pathway [83], providing new insights for clinical cancer immunotherapy (Table 1).

**Table 1:** Mitochondrial transfer in the tumor microenvironment

Cells	Mechanism of Mitochondrial Transfer	Mitochondrial Traffic		Functional Consequences in Recipient Cells
B16 ρ0 mouse metastatic melanoma and 4T1 ρ0 mouse metastatic breast cancer cells	Not clarified	Acquisition of mtDNA from the microenvironment		Respiratory function was restored and tumorigenicity enhanced [48]
Human MSCs and cancer cell lines	TNTs	Bidirectional between MSCs and cancer cells		Increased OXPHOS as well as enhanced invasion [42]
Human AML cell lines, umbilical CB and MS-5 stromal cell line	Endocytosis	Unidirectional from stromal cells to AML cells		Increased AML cells OXPHOS as well as chemotherapy resistance [76]
Human mesothelioma and benign mesothelial cell lines	TNTs	Bidirectional between malignant or between normal		Increased invasion [21]
Human 143B ρ0 osteosarcoma cells and human WJMSCs	Not clarified	Unidirectional from WJMSCs to 143B ρ0		Respiratory function was restored [47]
Rat PC12 phaeochromocytoma cells untreated ± UV-damage	TNTs—MPs	Unidirectional from healthy to UV-damaged cells		Rescued of UV-damaged from apoptosis [66]
Endothelial、MSCs and human ovarian and breast cancer cells	TNTs	Bidirectional (preference for endothelial to MSCs)	Chemotherapy resistance [18]	
Human mitochondria-deficient (ρ0) 143B ρ0 osteosarcoma cells and MSCs	TNTs	Unidirectional from MSCs to 143B ρ0	Restoration of mitochondrial functions [45]	
Human AML blasts and BMSCs, healthy hematopoietic stem cells	TNTs	Unidirectional from BMSCs to AML blasts but not to healthy hematopoietic stem cells	Promoted AML blasts proliferation [50]	
Human highly malignant T24 urothelial carcinoma cells and non-malignant RT4 urinary papillary urothelial cells	TNTs	Unidirectional from malignant to nonmalignant cells	Enhanced non-malignant cell invasiveness [12]	
Primary human multiple myeloma cells and cell lines, BMSCs	TNTs	Bidirectional	Increased proliferation and chemotherapy resistance multiple myeloma cells [73]	
Human MSCs and ALL cells	TNTs	Unidirectional from MSCs to ALL cells	Chemoprotection from ROS-induced therapy [15]	
Human LSCC	TNTs	Not clarified	No documented consequences [84]	
Human MSCs, Jurkat cells and T-ALL cells	TNTs	Bidirectional, but mostly from Jurkat cells to MSCs and from T-ALL cells to MSCs	Chemoresistance of Jurkat and T-ALL cells [23]	
Human TASCs and glioblastoma cells	TNTs, EVs and cannibalism	Unidirectional from TASCs to primary glioblastoma cells	Chemoresistance, Radiotherapy resistance,increased proliferation in glioblastoma cells [52]	
Human PC3 prostate cancer cells and CAFs	TNTs	Unidirectional from CAFs to PC3	Increased migratory and metastatic abilities of prostate cancer cells [14]	
MDA-MB-231 cells and hungry MDA-MB-231 cells	EVs	Unidirectional from MDA-MB-231 cells to hungry MDA-MB-231 cells	Increased invasiveness and metastasis [13]	
Lung cancer cells and CD8 + T cell	Endocytosis (CICs)	Unidirectional from lymphocyte to Lung cancer cells	Increased proliferation and immune escape [17]	
Cancer cells and endothelial cells	TNTs	Bidirectional between endothelial cells and cancer cells	Increased Chemotherapy resistance [18]	
Cancer cells and platelet	TNTs	Unidirectional from platelet to cancer cells	Promoted metastasis and reduced oxidative stress [20]	
Adipose stem cells and HEK293	TNTs	Unidirectional from Adipose stem cells to HEK293	Enhanced tumorigenicity [53]	
PCa cells and CAFs	TNTs	Unidirectional from CAFs to PCa cells	Increased invasiveness and metastasis [58]	
Chemotherapy-resistant TNBC cells and TNBC cells	TNTs	Unidirectional from Chemotherapy-resistant TNBC cells to TNBC cells	Increased Chemotherapy resistance [80]	
Melanoma cells and MSCs	TNTs	Unidirectional from MSCs to Melanoma cells	Increased proliferation [56]	

## Clinical application of mitochondrial transplantation

**Fig. 6:**
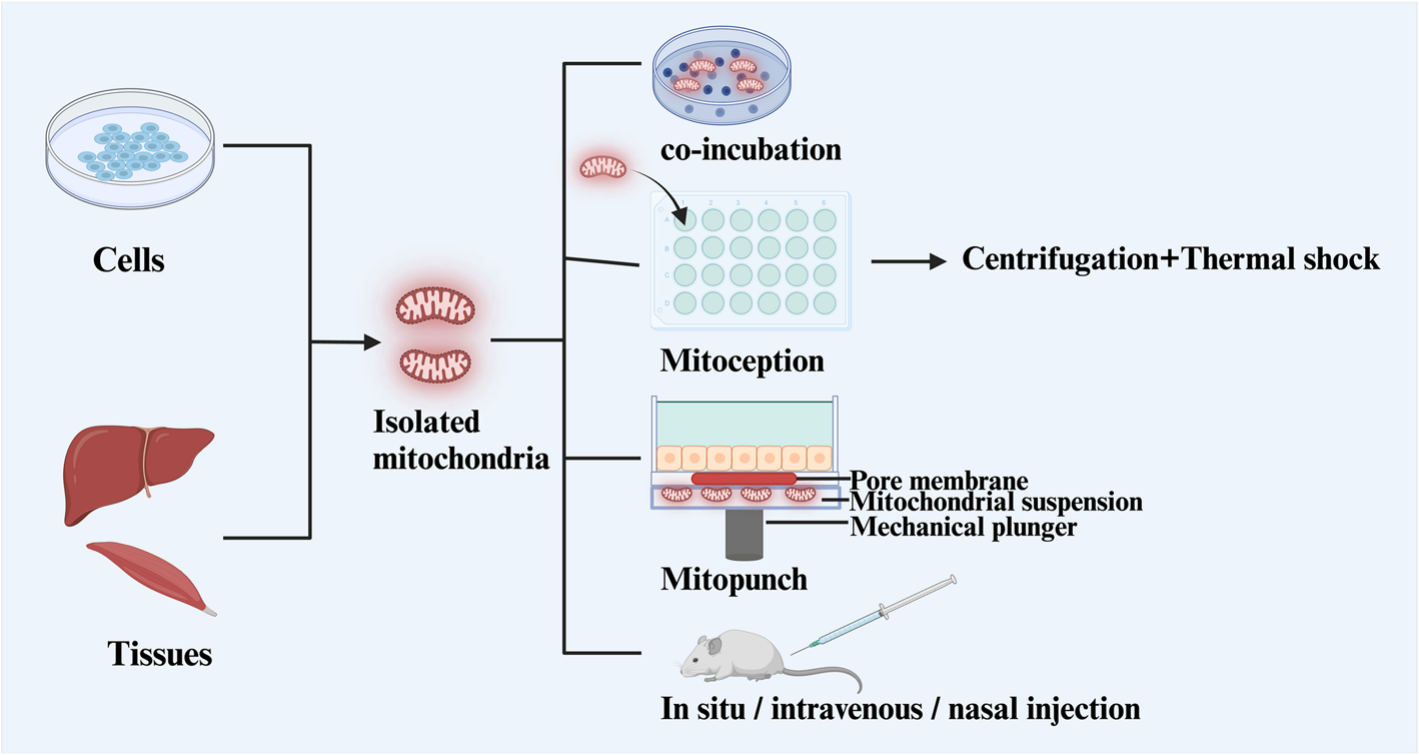
The process of mitochondrial transplantation. The process of mitochondrial transplantation involves the isolation of healthy mitochondria from cells or tissues. In *vitro* mitochondrial transplantation usually co-incubates the isolated mitochondria with recipient cells, and techniques such as Mitoception or Mitopunch are used to increase efficiency. In *vivo* mitochondrial transplantation can be directly administered through in situ injection, intravenous injection, or intranasal injection

With the revelation of intercellular mitochondrial transfer, mitochondrial transplantation based on this phenomenon is gradually increasing across various medical fields. Although mitochondrial transfer in the tumor microenvironment generally promotes tumor progression, introducing healthy mitochondria into cells exogenously can produce the opposite effect. At present, the first step in mitochondrial transplantation is to isolate functional mitochondria from cells and tissues. Several methods for mitochondrial transplantation in *vitro* already exist, with incubation being the most basic method, which involves co-culturing isolated mitochondria with recipient cells. However, this basic method is limited by the endocytosis capabilities of the recipient cells, so researchers have employed various methods to improve efficiency [85]. For example, MitoCeption mentioned before and Mitopunch, which uses a pressure-driven device to introduce isolated mitochondria into recipient cells lacking mtDNA (Fig. 6) [86]. Mitochondrial transplantation for oocytes can be done using autologous microinjection. Other than methods for mitochondrial transplantation in *vitro*, there are also methods for in *vivo* transplantation, including direct injection, intravenous injection, or intranasal injection [86].

### Apoptosis induced by mitochondrial transplantation

**Fig. 7:**
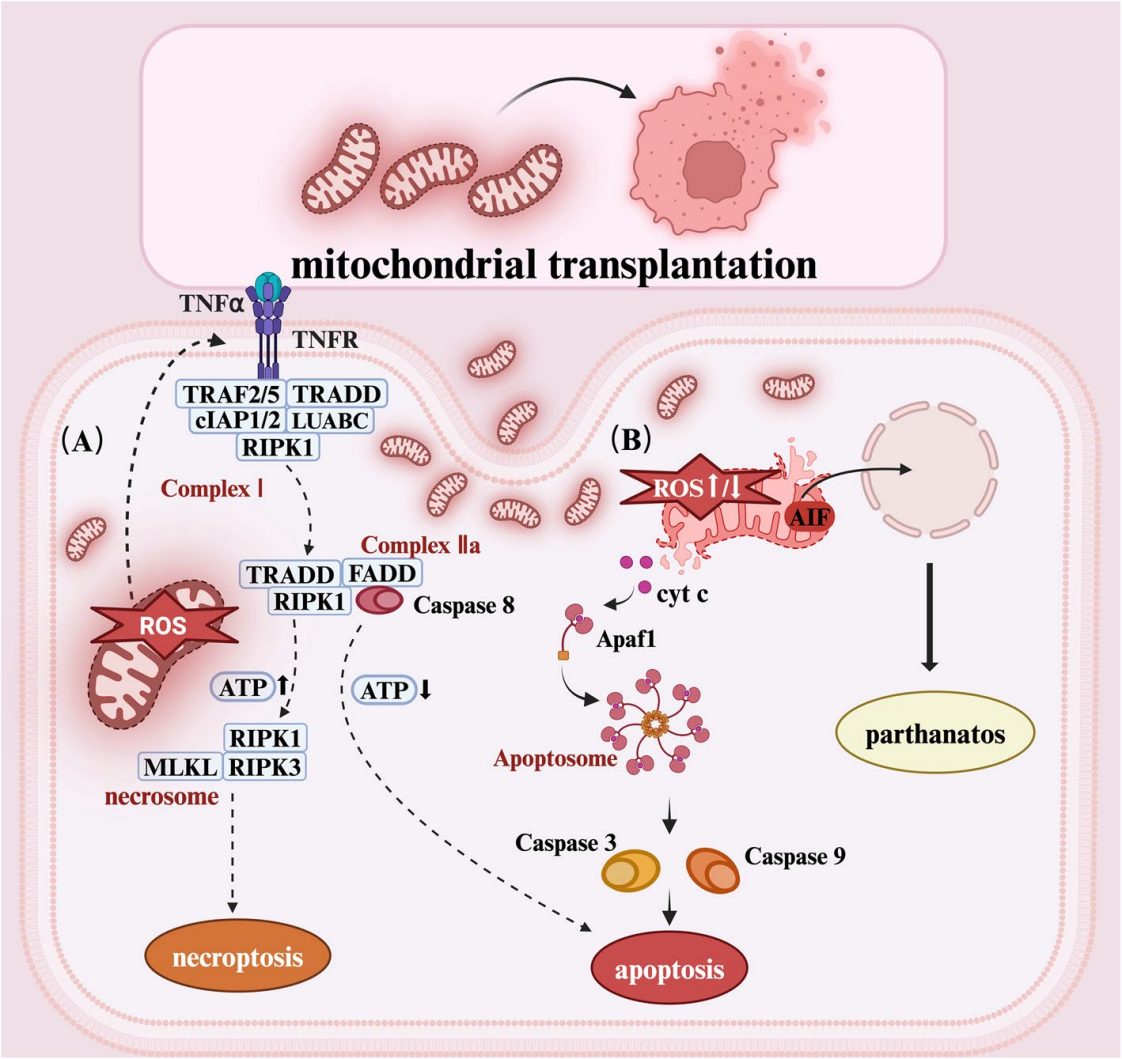
Mitochondrial transplantation leads to apoptosis, parthanatos, and necroptosis. **A** If the transplanted mitochondria lead to an increase in intracellular ROS, it stimulates the release of TNF-α, which then binds to tumor necrosis factor receptor (TNFR), triggering the recruitment of related proteins to form complex I, subsequently forming complex IIa, and eventually leading to the formation of the necrosome, causing necroptosis. **B** Transplanted mitochondria cause changes in intracellular ROS. Whether an increase or a decrease level of ROS, induces apoptosis through the release of cytC. In addition, AIF might also be released from the mitochondria into the nucleus during this process, causing parthanatos

Although mitochondrial transfer within the tumor microenvironment generally accelerates tumor development, introducing healthy mitochondria into cells exogenously triggers the opposite effect. For instance, implanting foreign mitochondria into tumor cells induces apoptosis in these cells. Researchers have found that transplanting mitochondria into DU145, PC3, or SKOV3 cancer cells, in combination with low-dose chemotherapy, significantly increased apoptosis in these cells, enhancing the sensitivity of mice to chemotherapy [87]. In tumor cells, the mitochondrial apoptosis system is suppressed while the anti-apoptosis system is activated, preventing tumor cells from undergoing apoptosis [88]. However, multiple studies on mitochondrial transplantation have found that after the transplant, the mitochondrial apoptosis pathway in tumor cells is reactivated, also enhancing the sensitivity of corresponding tumors to chemotherapy [87–90]. The transplantation of healthy mitochondria into cancer cells can induce apoptosis based on the production of ROS [91]. This is because different types of cancer have different needs for ROS, and even within the same tumor type, the need for ROS varies at different stages [92]. Therefore, both an increase or a decrease in ROS may activate the apoptotic signaling pathways. Changes in ROS prompt mitochondrial pores to release cytochrome C (cytC), thereby activating apoptotic protease activating factor 1 (Apaf1) to induce the formation of the apoptosome, which then activates Caspase 9 and Caspase 3, triggering apoptosis [91] (Fig. 7). By co-culturing HuCCT1 cells and mitochondria isolated from 143Bρ0 cells, it was found that mitochondrial transplantation promoted apoptosis in cholangiocarcinoma (CCA) cells, thereby inhibiting CCA cell proliferation. This process relied on the phosphatase and tensin homolog (PTEN)/Phosphoinositide 3-Kinase (PI3K)/AKT signaling pathway, where the production of ROS and mtROS significantly decreased in CCA cells after transplantation from 143Bρ0 cells, and PTEN expression was activated. As PTEN is a negative regulator of PI3K, it inhibits the expression of PI3K and its downstream molecule AKT [93]. The PI3K/AKT signaling pathway itself inhibits apoptosis, so with the downregulation of PI3K and AKT, the inhibition of apoptosis is weakened, thus increasing apoptosis in cancer cells and achieving a tumor-suppressive effect [94]. In experiments on mitochondrial transplantation in hepatocellular carcinoma, healthy mitochondria were found to inhibit tumor cells from undergoing glycolysis, promoted dephosphorylation of p-Bad, downregulated the expression of Bcl-2, increased Bax, and finally induced tumor cell apoptosis in a Caspase-dependent manner [95]. Under the mediation of Pep-1, transporting mitochondria to MCF-7 breast cancer cells decreased the viability of MCF-7 cells and induced caspase-independent and apoptosis-inducing factor (AIF)-mediated cell death [96], proving that mitochondrial transplantation can also induce other forms of cell death besides apoptosis. Under the stimulation of mitochondrial transplantation, the permeability of outer mitochondrial membrane changes [97]. At this time, AIF is released from mitochondria into the cytosol and then transported into the cell nucleus, promoting chromatin condensation and DNA degradation in a caspase-independent manner, causing a form of death known as parthanatos [98]. Moreover, in melanoma mouse models, it was found that the tumor-suppressive effect produced by importing healthy mitochondria from female sources was stronger than that from male sources [88], potentially indicating the greater therapeutic value of female mitochondria over male mitochondria in treating melanoma.

### Necroptosis induced by mitochondrial transplantation

Mitochondrial transplantation, in addition to inducing cancer cell apoptosis, can also lead to necroptosis under certain conditions. When the transplanted mitochondria increase ROS within cancer cells, the excess ROS stimulates the release of TNF-α, which then activates its corresponding receptors, triggering the recruitment of related proteins to the cell membrane [91]. These proteins include receptor-interacting protein kinase 1 (RIPK1), TNFR-associated death domain (TRADD), TNFR-associated factor 2 (TRAF2), TRAF5, cellular inhibitor of apoptosis 1 (cIAP1), cIAP2, and linear ubiquitin chain assembly complex (LUBAC). These proteins form complex I, from which TRADD and RIPK1 dissociate and undergo different processes to form two types of complex II, with complex IIa composed of FADD, TRADD, RIPK1, and caspase 8. In complex IIa, when the levels of RIPK3 and mixed lineage kinase domain-like (MLKL) are sufficient and caspase 8 is inactivated, RIPK3 is recruited [99]. Recruited RIPK3, RIPK1, and MLKL proteins form the necrosome [100], after which MLKL forms pores in the cell membrane, ultimately leading to cell necroptosis [91]. It is noteworthy that the appearance of caspase 8 in the process of necrosis means that this signaling pathway can simultaneously induce apoptosis. This is closely related to the intracellular ATP content; high ATP levels convert the cell fate towards apoptosis, while necroptosis, which occurs without ATP, is more likely to happen in a low ATP cellular environment [91]. Therefore, the therapeutic effect of mitochondrial transplantation in cancer cells may not only solely rely on apoptosis but also be associated with necroptosis and parthanatos.

### Blocking mitochondrial transfer with drugs

Although mitochondrial transplantation can exert a tumor-suppressing effect, a lot of work is needed before it can be practically applied in humans. Firstly, it concerns how to ensure that mitochondria overcome the assault of the high calcium environment outside the cell during transportation within the body [101], so as to target tumor tissues, and achieve an effective concentration that inhibits tumor growth within tumor cells. Secondly, whether different types of tumors can be treated with the same type of mitochondrial transplantation, or whether mitochondria transplanted to different types of tumors need to have relevant specificity [102]. In addition, if we want to apply this widely in clinical trials, effectively preserving mitochondria from degradation is also a challenge that must be overcome. Lastly, the safety issues of mitochondrial transplantation remain to be resolved; we still need more research to confirm the safety and feasibility of this treatment method.

Although the application of mitochondrial transplantation is currently limited, however, this does not mean we can not draw inspiration from mitochondrial transfer. As mentioned earlier, spontaneous mitochondrial transfer mostly promotes tumor development. Thus, designing drugs to reverse the transfer of mitochondria between tumor cells and their microenvironment could be a strategy to inhibit tumors [16]. Intercellular mitochondrial transfer is mediated by TNTs, extracellular vesicles, and gap junctions [103]. Inhibitors targeting gap junctions include 18-α-glycyrrhetinic acid, and for inhibiting vesicular endocytosis, dynasore, a dynamin inhibitor, can be used [23]. However, TNTs are the most common route for mitochondrial transfer [83], so blocking TNTs might be an effective therapeutic strategy. Taxanes and vinca alkaloids can inhibit mitochondrial transfer by preventing microtubule polymerization [34]. Additionally, actin polymerization inhibitors such as cytochalasin B (CytoB) [104], CytoD [23], and metformin also inhibit the formation of TNTs, thereby reducing mitochondrial transfer. In the experiment where tumor cells deprive T cells of mitochondria, researchers found that L-778,123 (an inhibitor of farnesyltransferase and geranylgeranyl transferase I) could inhibit the formation of nanotubes and mitochondrial transfer at non-cytotoxic concentrations [16]. Inhibiting nanotubes, which mediate tumor cells hijacking mitochondria of immune cells, is a new mechanism for tumor immune evasion. Therefore, using inhibitors of nanotubes to prevent tumor cells from hijacking T cell mitochondria can prevent immune cell exhaustion [16], which is crucial for restoring anti-tumor immunity. M-sec also serves as a therapeutic target. Inhibiting M-sec blocks the formation of TNTs [105]. Due to the lack of specific TNT markers, the inhibitors mentioned earlier, including L-778,123 and cytochalasins, only partially prohibit the formation of TNTs and have limited ability to inhibit tumor growth [83]. Future research should focus on specific inhibitors of TNTs as targets.

### Mitochondrial retransfusion promotes anti-tumor immunity

Chimeric antigen receptor (CAR) T-cell therapy represents a groundbreaking innovation in immune cell therapy, offering a personalized cancer immunotherapy approach. By modifying patients' T cells to express CARs—composed of antibodies and T cell receptors—on their surface, these cells can recognize tumor-associated antigens (TAAs) in an MHC-independent manner, thereby inducing the identification and destruction of tumor cells [106, 107]. While CAR-T cell therapy has shown good efficacy in treating hematological tumors, its effectiveness against solid tumors has been less satisfactory [108, 109]. Moreover, treatment failures have also been observed in some patients with hematological malignancies following CAR-T cell therapy [110]. A major limiting factor in the therapeutic efficacy of CAR-T cell therapy is the early exhaustion of T cells, characterized by impaired mitochondrial function and dynamics [109].

T cells, as crucial players in the anti-tumor immune response, rely on their mitochondrial activity at various stages when generating an immune response. From T cell polarization and migration, the formation of immune synapses, to T cell activation, proliferation, and differentiation, mitochondria play an essential and diverse role. Mitochondria are not only producers of ATP but also participate in calcium homeostasis regulation, lipid synthesis, and cell apoptosis, and other processes [111]. Therefore, targeting mitochondria could be a strategy to avoid T cell exhaustion in CAR-T cell therapy. Mitochondrial transplantation increases the mitochondrial content in T cells, further enhancing T cell function. By serving as a positive regulator, "supercharging" T cells with mitochondria makes them more effective at attacking tumor cells [110]. Research has already demonstrated the feasibility of transplanting mitochondria from human dermal fibroblasts (NHDF Neo) to human Jurkat cells (immortalized T cells) [102]. Furthermore, researchers have transferred prepared Q-mitochondria into CAR-T cells. The results demonstrated that this led to improved metabolic adaptability, enhanced proliferation capacity and cytokine production ability, and strengthened anti-tumor capability in CAR-T cells [110], potentially boosting the therapeutic effects of CAR-T treatment. These findings suggest that mitochondrial transplantation has the potential to complement CAR-T cell therapy and can be combined with other pharmacological methods to further enhance the effectiveness of CAR-T cell therapy.

## Conclusions

In modern oncology research, there is a pressing need to deepen our understanding on tumor metabolic reprogramming and the mechanisms behind mitochondrial transfer. This interest is not just about unraveling the fundamental mechanisms of tumor cell survival and proliferation; it also refers to the complexity of immunotherapy strategies and the challenges they face. Through metabolic reprogramming, tumor cells exhibit adaptability to internal and external environmental stresses, a capability that is particularly evident in their interactions with surrounding cells, especially during the process of mitochondrial transfer. This phenomenon not only adds complexity to the tumor metabolic network but also reveals the intricate interplay between the tumor and its microenvironment. Understanding this interplay is crucial for grasping how tumors survive and proliferate under adverse conditions.

Utilizing nanotechnology and single-cell analysis, scientists can now explore these biological processes with unprecedented precision, deepening our understanding on tumor cell metabolic pathways. This progress not only facilitates the study of intercellular interactions and communication mechanisms but also inspires the development of new therapeutic approaches. However, despite the new research directions opened by these technologies, our understanding of the molecular mechanisms behind mitochondrial transfer, particularly how they exhibit uniqueness across different tissues and cell types, remains limited. If the specific molecular mechanisms by which mitochondrial transfer promotes tumor development, including proliferation, invasion and metastasis, chemotherapy resistance, immune evasion and so on, could be clarified, it would provide numerous targets for drug research. Further research in this area will be key to achieving more personalized and precise cancer treatment strategies.

Mitochondrial transplantation therapy for tumor is still in the experimental stage, although showing potential therapeutic effects in animal models, yet it has not progressed to clinical use. The transition from laboratory research to clinical application presents multiple challenges, such as safety concerns, the targeting of mitochondria to tumor cells, and preservation issues. Future research must overcome these hurdles to successfully translate these findings into clinical benefits for cancer patients. Meanwhile, current research is focusing on how to block the pathways of mitochondrial transfer between tumor cells. The preliminary success of this strategy gives us hope, suggesting that by intervening in the metabolic interactions of tumor cells, we might develop new therapeutic approaches to inhibit tumor growth and spread.

The application of mitochondrial transfer in immunotherapy, especially in CAR-T cell therapy, has begun to show its unique advantages. By enhancing the metabolic capacity of T cells, mitochondrial transplantation not only increases the activity of CAR-T cells against tumors but also helps these cells survive in the inhibitory tumor microenvironment, thereby improving the effectiveness of the treatment. This progress not only brings new hope to the field of immunotherapy but also further emphasizes the importance of understanding and utilizing the metabolic checkpoints of tumor cells in cancer treatment.

Moreover, existing research has also demonstrated that transferring mitochondria into tumor cells artificially can promote tumor growth, a finding that seems contradictory to other studies where mitochondrial transplantation was shown to suppress tumor development. This raises the question: whether mitochondrial transplantation possesses a dual nature, or whether it only suppresses tumor growth under specific conditions? Additionally, the concentration of transferred mitochondria appears to influence the extent of tumor promotion. This relationship is not straightforwardly proportional; rather, it is when mitochondria are at moderate concentrations that tumor growth is most significantly enhanced. Could this imply that excessively high concentrations of mitochondria might inhibit tumor cells? These are crucial questions that merit further investigation.

In summary, the in-depth study of tumor cell metabolic reprogramming and the mechanisms of mitochondrial transfer not only showcases the adaptability and biological flexibility of tumors but also provides an important scientific foundation for overcoming the limitations of current treatment methods and designing new therapeutic strategies. Based on this, we have reason to believe that further exploration of these complex biological processes will be able to offer more personalized and precise treatment options for tumor patients.

## Data Availability

Not applicable.

## Abbreviations

AML: Acute myeloid leukemia
HK: Hexokinase
PKM2: Pyruvate kinase M2
GLUT-1: Glucose transporter 1
AKT: Protein kinase B
PFK: Phosphofructokinase
mtDNA: Mitochondrial DNA
s-EVs: Small extracellular vesicles
TNTs: Tunneling nanotubes
PC: Pheochromocytoma
MSCs: Mesenchymal stem cells
SEM: Scanning electron microscopy
AFM: Atomic force microscopy
Miro1: Mitochondrial Rho GTPase 1
TRAK1: Transport associated protein 1
Myo19: Myosin XIX
KLF5: Kruppel-like factor 5
EVs: Extracellular vesicles
miRNA: microRNA
FACS: Fluorescence-activated cell sorting
qPCR: Real-time quantitative PCR
CytoD: Cytochalasin D
DRP1: Dynamin-related protein 1
FIS1: Fission 1 protein
NOX2: NADPH oxidase 2
BMSCs: Bone marrow stromal cells
ROS: Reactive Oxygen Species
TASCs: Tumor-associated stromal cells
GBM: Glioblastoma
CIC: Cancer-initiating cell
MAPK: Mitogen-activated protein kinase
MSCs: Marrow-derived stromal cells
mtROS: Mitochondrial Reactive Oxygen Species
TGF-β: Transforming growth factor- β
CAFs: Cancer-associated fibroblasts
MCT1: Monocarboxylate transporter 1
OXPHOS: Oxidative phosphorylation
MFF: Mitochondrial fission factor
EMT: Epithelial-mesenchymal transition
PINK1: PTEN-induced putative kinase 1
TLR9: Toll-like receptor 9
ETC: Electron transport chain
UV: Ultraviolet
AraC: Cytarabine
DNR: Daunorubicin
MMAF: Monomethyl auristatin-F
VCR: Vincristine
BM: Bone marrow
TNBC: Triple-negative breast cancer
CICs: Cell-in-cell structures
MERCI: Mitochondrial-enabled reconstruction of cellular interactions
TNF-α: Tumor necrosis factor-α
cytC: Cytochrome C
Apaf1: Apoptotic protease activating factor 1
CCA: Cholangiocarcinoma
PTEN: Phosphatase and tensin homolog
PI3K: Phosphoinositide 3-Kinase
AIF: Apoptosis-inducing factor
TNFR: Tumor necrosis factor receptor
RIPK1: Receptor-interacting protein kinase 1
TRADD: TNFR-associated death domain
TRAF2: TNFR-associated factor 2
cIAP1: Cellular inhibitor of apoptosis 1
LUBAC: Linear ubiquitin chain assembly complex
MLKL: Mixed lineage kinase domain-like
CytoB: Cytochalasin B
CAR: Chimeric antigen receptor
TAAs: Tumor-associated antigens
WJMSCs: Wharton’s jelly mesenchymal stem cells
MPs: Membrane protrusions
ALL: Acute lymphoblastic leukemia
LSCC: Laryngeal squamous cell carcinoma

## References

[1] Hanahan D, Weinberg RA (2011). Hallmarks of Cancer: The Next Generation. Cell.

[2] Koppenol WH, Bounds PL, Dang CV (2011). Otto Warburg’s contributions to current concepts of cancer metabolism. Nat Rev Cancer.

[3] Nunnari J, Suomalainen A (2012). Mitochondria. Cell.

[4] Lee J, Yesilkanal AE, Wynne JP (2019). Effective breast cancer combination therapy targeting BACH1 and mitochondrial metabolism. Nature.

[5] Murakami S, Nemazanyy I, White SM (2019). A Yap-Myc-Sox2-p53 Regulatory Network Dictates Metabolic Homeostasis and Differentiation in Kras-Driven Pancreatic Ductal Adenocarcinomas. Dev Cell.

[6] Tang YC, Hsiao JR, Jiang SS (2021). c-MYC-directed NRF2 drives malignant progression of head and neck cancer via glucose-6-phosphate dehydrogenase and transketolase activation. Theranostics.

[7] Cheng SC, Quintin J, Cramer RA (2014). mTOR- and HIF-1α–mediated aerobic glycolysis as metabolic basis for trained immunity. Science.

[8] Dang L, White DW, Gross S (2009). Cancer-associated IDH1 mutations produce 2-hydroxyglutarate. Nature.

[9] Wise DR, Ward PS, Shay JES (2011). Hypoxia promotes isocitrate dehydrogenase-dependent carboxylation of α-ketoglutarate to citrate to support cell growth and viability. Proc Natl Acad Sci.

[10] Fendt SM, Bell EL, Keibler MA (2013). Reductive glutamine metabolism is a function of the α-ketoglutarate to citrate ratio in cells. Nat Commun.

[11] Borcherding N, Brestoff JR (2023). The power and potential of mitochondria transfer. Nature.

[12] Lu J, Zheng X, Li F (2017). Tunneling nanotubes promote intercellular mitochondria transfer followed by increased invasiveness in bladder cancer cells. Oncotarget.

[13] Rabas N, Palmer S, Mitchell L (2021). PINK1 drives production of mtDNA-containing extracellular vesicles to promote invasiveness. J Cell Biol.

[14] Ippolito L, Morandi A, Taddei ML (2019). Cancer-associated fibroblasts promote prostate cancer malignancy via metabolic rewiring and mitochondrial transfer. Oncogene.

[15] Burt R, Dey A, Aref S (2019). Activated stromal cells transfer mitochondria to rescue acute lymphoblastic leukemia cells from oxidative stress. Blood.

[16] Saha T, Dash C, Jayabalan R (2022). Intercellular nanotubes mediate mitochondrial trafficking between cancer and immune cells. Nat Nanotechnol.

[17] Wang S, Liu B, Huang J, He H, Li L, Tao A (2023). Cell-in-cell promotes lung cancer malignancy by enhancing glucose metabolism through mitochondria transfer. Exp Cell Res.

[18] Pasquier J, Guerrouahen BS, Al Thawadi H (2013). Preferential transfer of mitochondria from endothelial to cancer cells through tunneling nanotubes modulates chemoresistance. J Transl Med.

[19] Valdebenito S, Malik S, Luu R (2021). Tunneling nanotubes, TNT, communicate glioblastoma with surrounding non-tumor astrocytes to adapt them to hypoxic and metabolic tumor conditions. Sci Rep.

[20] Zhang W, Zhou H, Li H (2023). Cancer cells reprogram to metastatic state through the acquisition of platelet mitochondria. Cell Rep.

[21] Lou E, Fujisawa S, Morozov A (2012). Tunneling Nanotubes Provide a Unique Conduit for Intercellular Transfer of Cellular Contents in Human Malignant Pleural Mesothelioma. Yang PC, ed.. PLoS ONE.

[22] You R, Wang B, Chen P (2022). Metformin sensitizes AML cells to chemotherapy through blocking mitochondrial transfer from stromal cells to AML cells. Cancer Lett.

[23] Wang J, Liu X, Qiu Y (2018). Cell adhesion-mediated mitochondria transfer contributes to mesenchymal stem cell-induced chemoresistance on T cell acute lymphoblastic leukemia cells. J Hematol OncolJ Hematol Oncol.

[24] Takenaga K, Koshikawa N, Nagase H (2021). Intercellular transfer of mitochondrial DNA carrying metastasis-enhancing pathogenic mutations from high- to low-metastatic tumor cells and stromal cells via extracellular vesicles. BMC Mol Cell Biol.

[25] Austefjord MW, Gerdes HH, Wang X (2014). Tunneling nanotubes: Diversity in morphology and structure. Commun Integr Biol.

[26] Liu Z, Sun Y, Qi Z, Cao L, Ding S (2022). Mitochondrial transfer/transplantation: an emerging therapeutic approach for multiple diseases. Cell Biosci.

[27] Yang F, Zhang Y, Liu S (2022). Tunneling Nanotube-Mediated Mitochondrial Transfer Rescues Nucleus Pulposus Cells from Mitochondrial Dysfunction and Apoptosis. Zhao FD, ed.. Oxid Med Cell Longev.

[28] Yang C, Endoh M, Tan DQ (2021). Mitochondria transfer from early stages of erythroblasts to their macrophage niche via tunnelling nanotubes. Br J Haematol.

[29] Rustom A, Saffrich R, Markovic I, Walther P, Gerdes HH (2004). Nanotubular Highways for Intercellular Organelle Transport. Science.

[30] Plotnikov EY, Khryapenkova TG, Vasileva AK (2008). Cell-to-cell cross-talk between mesenchymal stem cells and cardiomyocytes in co-culture. J Cell Mol Med.

[31] Ahani E, Fereydouni M, Motaghed M, Kepley CL (2022). Identification and Characterization of Tunneling Nanotubes Involved in Human Mast Cell FcεRI-Mediated Apoptosis of Cancer Cells. Cancers.

[32] Sahinbegovic H, Jelinek T, Hrdinka M (2020). Intercellular Mitochondrial Transfer in the Tumor Microenvironment. Cancers.

[33] Guo X, Can C, Liu W (2023). Mitochondrial transfer in hematological malignancies. Biomark Res.

[34] Zampieri LX, Silva-Almeida C, Rondeau JD, Sonveaux P (2021). Mitochondrial Transfer in Cancer: A Comprehensive Review. Int J Mol Sci.

[35] Domhan S, Ma L, Tai A (2011). Intercellular Communication by Exchange of Cytoplasmic Material via Tunneling Nano-Tube Like Structures in Primary Human Renal Epithelial Cells. PLoS ONE..

[36] Ahmad T, Mukherjee S, Pattnaik B (2014). Miro1 regulates intercellular mitochondrial transport enhances mesenchymal stem cell rescue efficacy.

[37] Meng W, He C, Hao Y, Wang L, Li L, Zhu G (2020). Prospects and challenges of extracellular vesicle-based drug delivery system: considering cell source. Drug Deliv.

[38] Zappulli V, Friis KP, Fitzpatrick Z, Maguire CA, Breakefield XO (2016). Extracellular vesicles and intercellular communication within the nervous system. J Clin Invest.

[39] Berridge MV, McConnell MJ, Grasso C, Bajzikova M, Kovarova J, Neuzil J (2016). Horizontal transfer of mitochondria between mammalian cells: beyond co-culture approaches. Curr Opin Genet Dev.

[40] Nzigou Mombo B, Gerbal-Chaloin S, Bokus A (2017). MitoCeption: Transferring Isolated Human MSC Mitochondria to Glioblastoma Stem Cells. J Vis Exp.

[41] Cabrera F, Ortega M, Velarde F (2019). Primary allogeneic mitochondrial mix (PAMM) transfer/transplant by MitoCeption to address damage in PBMCs caused by ultraviolet radiation. BMC Biotechnol.

[42] Caicedo A, Fritz V, Brondello JM (2015). MitoCeption as a new tool to assess the effects of mesenchymal stem/stromal cell mitochondria on cancer cell metabolism and function. Sci Rep.

[43] Masuzawa A, Black KM, Pacak CA (2013). Transplantation of autologously derived mitochondria protects the heart from ischemia-reperfusion injury. Am J Physiol-Heart Circ Physiol.

[44] Pacak CA, Preble JM, Kondo H (2015). Actin-dependent mitochondrial internalization in cardiomyocytes: evidence for rescue of mitochondrial function. Biol Open.

[45] Cho YM, Kim JH, Kim M (2012). Mesenchymal Stem Cells Transfer Mitochondria to the Cells with Virtually No Mitochondrial Function but Not with Pathogenic mtDNA Mutations. Moran M, ed.. PLoS ONE.

[46] Dong LF, Kovarova J, Bajzikova M (2017). Horizontal transfer of whole mitochondria restores tumorigenic potential in mitochondrial DNA-deficient cancer cells. eLife..

[47] Lin HY, Liou CW, Chen SD (2015). Mitochondrial transfer from Wharton’s jelly-derived mesenchymal stem cells to mitochondria-defective cells recaptures impaired mitochondrial function. Mitochondrion.

[48] Tan AS, Baty JW, Dong LF (2015). Mitochondrial Genome Acquisition Restores Respiratory Function and Tumorigenic Potential of Cancer Cells without Mitochondrial DNA. Cell Metab.

[49] Griessinger E, Moschoi R, Biondani G, Peyron JF (2017). Mitochondrial Transfer in the Leukemia Microenvironment. Trends Cancer.

[50] Marlein CR, Zaitseva L, Piddock RE (2017). NADPH oxidase-2 derived superoxide drives mitochondrial transfer from bone marrow stromal cells to leukemic blasts. Blood.

[51] Kidwell CU, Casalini JR, Pradeep S (2023). Transferred mitochondria accumulate reactive oxygen species, promoting proliferation. eLife..

[52] Salaud C, Alvarez-Arenas A, Geraldo F (2020). Mitochondria transfer from tumor-activated stromal cells (TASC) to primary Glioblastoma cells. Biochem Biophys Res Commun.

[53] Burch SA, Luna LC (2022). Effects of Cell Density and Microenvironment on Stem Cell Mitochondria Transfer among Human Adipose-Derived Stem Cells and HEK293 Tumorigenic Cells. Int J Mol Sci.

[54] Watson DC, Bayik D, Storevik S (2023). GAP43-dependent mitochondria transfer from astrocytes enhances glioblastoma tumorigenicity. Nat Cancer.

[55] Pinto G, Saenz-de-Santa-Maria I, Chastagner P (2021). Patient-derived glioblastoma stem cells transfer mitochondria through tunneling nanotubes in tumor organoids. Biochem J.

[56] Kumar PR, Saad M, Hellmich C (2022). PGC-1α induced mitochondrial biogenesis in stromal cells underpins mitochondrial transfer to melanoma. Br J Cancer.

[57] Fiaschi T, Marini A, Giannoni E (2012). Reciprocal Metabolic Reprogramming through Lactate Shuttle Coordinately Influences Tumor-Stroma Interplay. Cancer Res.

[58] Brauer HA, Makowski L, Hoadley KA (2013). Impact of Tumor Microenvironment and Epithelial Phenotypes on Metabolism in Breast Cancer. Clin Cancer Res.

[59] Giannoni E, Taddei ML, Morandi A (2015). Targeting stromal-induced pyruvate kinase M2 nuclear translocation impairs OXPHOS and prostate cancer metastatic spread. Oncotarget.

[60] Sotgia F, Whitaker-Menezes D, Martinez-Outschoorn UE (2012). Mitochondrial metabolism in cancer metastasis: Visualizing tumor cell mitochondria and the “reverse Warburg effect” in positive lymph node tissue. Cell Cycle.

[61] Guido C, Whitaker-Menezes D, Lin Z, et al. Mitochondrial fission induces glycolytic reprogramming in cancer-associated myofibroblasts, driving stromal lactate production, and early tumor growth. Oncotarget. 2012;3(8):798–810. 10.18632/oncotarget.574. 10.18632/oncotarget.574 PMC3478457 22878233

[62] Goliwas KF, Libring S, Berestesky E (2023). Mitochondrial transfer from cancer-associated fibroblasts increases migration in aggressive breast cancer. J Cell Sci..

[63] Kheirandish-Rostami M, Roudkenar MH, Jahanian-Najafabadi A (2020). Mitochondrial characteristics contribute to proliferation and migration potency of MDA-MB-231 cancer cells and their response to cisplatin treatment. Life Sci.

[64] Spees JL, Olson SD, Whitney MJ, Prockop DJ (2006). Mitochondrial transfer between cells can rescue aerobic respiration. Proc Natl Acad Sci.

[65] Porporato PE, Payen VL, Pérez-Escuredo J (2014). A Mitochondrial Switch Promotes Tumor Metastasis. Cell Rep.

[66] Wang X, Gerdes HH (2015). Transfer of mitochondria via tunneling nanotubes rescues apoptotic PC12 cells. Cell Death Differ.

[67] Cara S, Tannock IF (2001). Retreatment of patients with the same chemotherapy: Implications for clinical mechanisms of drug resistance. Ann Oncol.

[68] Dias MP, Moser SC, Ganesan S, Jonkers J (2021). Understanding and overcoming resistance to PARP inhibitors in cancer therapy. Nat Rev Clin Oncol.

[69] Fujiwara S, Kawano Y, Yuki H (2013). PDK1 inhibition is a novel therapeutic target in multiple myeloma. Br J Cancer.

[70] Dalva-Aydemir S, Bajpai R, Martinez M (2015). Targeting the Metabolic Plasticity of Multiple Myeloma with FDA-Approved Ritonavir and Metformin. Clin Cancer Res.

[71] Sanchez WY, McGee SL, Connor T (2013). Dichloroacetate inhibits aerobic glycolysis in multiple myeloma cells and increases sensitivity to bortezomib. Br J Cancer.

[72] Matula Z, Mikala G, Lukácsi S (2021). Stromal Cells Serve Drug Resistance for Multiple Myeloma via Mitochondrial Transfer: A Study on Primary Myeloma and Stromal Cells. Cancers.

[73] Marlein CR, Piddock RE, Mistry JJ (2019). CD38-Driven Mitochondrial Trafficking Promotes Bioenergetic Plasticity in Multiple Myeloma. Cancer Res.

[74] Matula Z, Uher F, Vályi-Nagy I, Mikala G (2023). The Effect of Belantamab Mafodotin on Primary Myeloma-Stroma Co-Cultures: Asymmetrical Mitochondrial Transfer between Myeloma Cells and Autologous Bone Marrow Stromal Cells. Int J Mol Sci.

[75] Kantarjian HM, O’Brien S, Smith TL (2000). Results of Treatment With Hyper-CVAD, a Dose-Intensive Regimen, in Adult Acute Lymphocytic Leukemia. J Clin Oncol.

[76] Moschoi R, Imbert V, Nebout M (2016). Protective mitochondrial transfer from bone marrow stromal cells to acute myeloid leukemic cells during chemotherapy. Blood.

[77] Saito K, Zhang Q, Yang H (2021). Exogenous mitochondrial transfer and endogenous mitochondrial fission facilitate AML resistance to OxPhos inhibition. Blood Adv.

[78] Farge T, Saland E, De Toni F (2017). Chemotherapy-Resistant Human Acute Myeloid Leukemia Cells Are Not Enriched for Leukemic Stem Cells but Require Oxidative Metabolism. Cancer Discov.

[79] Nakhle J, Khattar K, Özkan T (2023). Mitochondria Transfer from Mesenchymal Stem Cells Confers Chemoresistance to Glioblastoma Stem Cells through Metabolic Rewiring. Cancer Res Commun.

[80] Abad E, Lyakhovich A (2022). Movement of Mitochondria with Mutant DNA through Extracellular Vesicles Helps Cancer Cells Acquire Chemoresistance. ChemMedChem.

[81] Sansone P, Savini C, Kurelac I (2017). Packaging and transfer of mitochondrial DNA via exosomes regulate escape from dormancy in hormonal therapy-resistant breast cancer. Proc Natl Acad Sci..

[82] Choe YJ, Min JY, Lee HS (2022). Heterotypic cell-in-cell structures between cancer and NK cells are associated with enhanced anticancer drug resistance. iScience..

[83] Zhang H, Yu X, Ye J (2023). Systematic investigation of mitochondrial transfer between cancer cells and T cells at single-cell resolution. Cancer Cell.

[84] Antanavičiūtė I, Rysevaitė K, Liutkevičius V (2014). Long-Distance Communication between Laryngeal Carcinoma Cells. Scemes E, ed.. PLoS ONE.

[85] Jain R, Begum N, Tryphena KP (2023). Inter and intracellular mitochondrial transfer: Future of mitochondrial transplant therapy in Parkinson’s disease. Biomed Pharmacother.

[86] Sercel AJ, Patananan AN, Man T (2021). Stable transplantation of human mitochondrial DNA by high-throughput, pressurized isolated mitochondrial delivery. eLife..

[87] Celik A, Orfany A, Dearling J, Del Nido PJ, McCully JD, Bakar-Ates F (2023). Mitochondrial transplantation: Effects on chemotherapy in prostate and ovarian cancer cells in vitro and in vivo. Biomed Pharmacother.

[88] Yu Z, Hou Y, Zhou W, Zhao Z, Liu Z, Fu A (2021). The effect of mitochondrial transplantation therapy from different gender on inhibiting cell proliferation of malignant melanoma. Int J Biol Sci.

[89] Elliott RL, Jiang XP, Head JF (2012). Mitochondria organelle transplantation: introduction of normal epithelial mitochondria into human cancer cells inhibits proliferation and increases drug sensitivity. Breast Cancer Res Treat.

[90] Sun C, Liu X, Wang B (2019). Endocytosis-mediated mitochondrial transplantation: Transferring normal human astrocytic mitochondria into glioma cells rescues aerobic respiration and enhances radiosensitivity. Theranostics.

[91] Cruz-Gregorio A, Aranda-Rivera AK, Amador-Martinez I, Maycotte P (2023). Mitochondrial transplantation strategies in multifaceted induction of cancer cell death. Life Sci.

[92] Cheung EC, DeNicola GM, Nixon C (2020). Dynamic ROS Control by TIGAR Regulates the Initiation and Progression of Pancreatic Cancer. Cancer Cell.

[93] Liu X, Zhang Y, Yang X (2023). Mitochondrial transplantation inhibits cholangiocarcinoma cells growth by balancing oxidative stress tolerance through PTEN/PI3K/AKT signaling pathway. Tissue Cell.

[94] Braglia L, Zavatti M, Vinceti M, Martelli AM, Marmiroli S (2020). Deregulated PTEN/PI3K/AKT/mTOR signaling in prostate cancer: Still a potential druggable target?. Biochim Biophys Acta BBA - Mol Cell Res.

[95] Zhou W, Zhao Z, Yu Z, Hou Y, Keerthiga R, Fu A (2022). Mitochondrial transplantation therapy inhibits the proliferation of malignant hepatocellular carcinoma and its mechanism. Mitochondrion.

[96] Chang JC, Chang HS, Wu YC (2019). Mitochondrial transplantation regulates antitumour activity, chemoresistance and mitochondrial dynamics in breast cancer. J Exp Clin Cancer Res.

[97] Huang P, Chen G, Jin W, Mao K, Wan H, He Y (2022). Molecular Mechanisms of Parthanatos and Its Role in Diverse Diseases. Int J Mol Sci.

[98] Gupta S, Kass GEN, Szegezdi E, Joseph B (2009). The mitochondrial death pathway: a promising therapeutic target in diseases. J Cell Mol Med.

[99] Chen J, Kos R, Garssen J, Redegeld F (2019). Molecular Insights into the Mechanism of Necroptosis: The Necrosome as a Potential Therapeutic Target. Cells.

[100] Grootjans S, Vanden Berghe T, Vandenabeele P (2017). Initiation and execution mechanisms of necroptosis: an overview. Cell Death Differ.

[101] Mitochondrial transplantation in humans: “magical” cure or cause for concern? - PubMed. Accessed 26 Apr 2024. https://pubmed.ncbi.nlm.nih.gov/30371508/10.1172/JCI124944 PMC6264628 30371508

[102] Hosseinian S, Ali Pour P, Kheradvar A (2022). Prospects of mitochondrial transplantation in clinical medicine: Aspirations and challenges. Mitochondrion.

[103] Hsu YC, Wu YT, Yu TH, Wei YH (2016). Mitochondria in mesenchymal stem cell biology and cell therapy: From cellular differentiation to mitochondrial transfer. Semin Cell Dev Biol.

[104] Mohammadalipour A, Dumbali SP, Wenzel PL (2020). Mitochondrial Transfer and Regulators of Mesenchymal Stromal Cell Function and Therapeutic Efficacy. Front Cell Dev Biol.

[105] Clemente-Suárez VJ, Martín-Rodríguez A, Yáñez-Sepúlveda R, Tornero-Aguilera JF (2023). Mitochondrial Transfer as a Novel Therapeutic Approach in Disease Diagnosis and Treatment. Int J Mol Sci.

[106] Rad SMAH, Halpin JC, Mollaei M, Smith Bell SWJ, Hirankarn N, McLellan AD (2021). Metabolic and Mitochondrial Functioning in Chimeric Antigen Receptor (CAR)—T Cells. Cancers.

[107] Lu J, Jiang G (2022). The journey of CAR-T therapy in hematological malignancies. Mol Cancer.

[108] Rostamian H, Khakpoor-Koosheh M, Fallah-Mehrjardi K, Mirzaei HR, Brown CE (2021). Mitochondria as Playmakers of CAR T-cell Fate and Longevity. Cancer Immunol Res.

[109] Huang Y, Si X, Shao M, Teng X, Xiao G, Huang H (2022). Rewiring mitochondrial metabolism to counteract exhaustion of CAR-T cells. J Hematol OncolJ Hematol Oncol.

[110] Harada S, Hashimoto D, Senjo H (2022). Intercellular Mitochondrial Transfer Enhances Metabolic Fitness and Anti-Tumor Effects of CAR T Cells. Blood.

[111] Desdín-Micó G, Soto-Heredero G, Mittelbrunn M (2018). Mitochondrial activity in T cells. Mitochondrion.

